# H_2_O_2_-modified NiO_x_ for perovskite photovoltaic modules

**DOI:** 10.1016/j.xinn.2024.100650

**Published:** 2024-05-27

**Authors:** Jianlong Xie, Qiyin Chen, Qin Xue, Igor F. Perepichka, Guohua Xie

**Affiliations:** 1 Hubei Key Lab on Organic and Polymeric Optoelectronic Materials, Department of Chemistry, Wuhan University, Wuhan 430072, China; 2 The Institute of Flexible Electronics (Future Technologies), Xiamen University, Xiamen 361005, China; 3 Department of Physical Science and Technology, Central China Normal University, Wuhan 430079, China; 4 Department of Physical Chemistry and Technology of Polymers, Silesian University of Technology, M. Strzody 9, 44-100 Gliwice, Poland; 5 Centre for Organic and Nanohybrid Electronics, Silesian University of Technology, S. Konarskiego 22b, 44-100 Gliwice, Poland

## Main text

Metal halide perovskite semiconductors have garnered interest as promising materials for solar cells due to their exceptional optoelectronic properties such as long carrier diffusion length, low cost, and solution processability. Notably, the power conversion efficiency (*PCE*) of single-junction perovskite solar cells (PSCs) has achieved a record-high value of 25.6%, while that of tandem PCSs reached nearly 30%.^1^ However, enhancing efficiency and ensuring long-term operational stability continue to pose significant challenge for PSCs. It is widely recognized that interfacial passivation could reduce interfacial charge recombination, enhance the crystalline quality of perovskites, and thus promote efficient hole collection.

## Interface engineering

As one of the most attractive inorganic p-type semiconductors in PSCs, NiO_x_ is a promising candidate for efficient hole transport layers (HTLs) due to its excellent properties such as high optical transmittance, easy processability, wide band gap, good energy alignment, and high thermal stability with perovskites. So far, various fabrication processes have been developed for the fabrication of NiO_x_ films, including sol-gel spin coating, electrodeposition, sputtering, etc. Among these methods, sol-gel spin coating is the one of the simplest and most cost-efficient ways to produce NiO_x_ films. It involves fabricating films from a nickel acetate solution and the subsequent annealing of the films in an ambient atmosphere. Yang’s group employed this solution-processing technique to create PSCs with NiO_x_ as the HTL, resulting in an open-circuit voltage (*V*_OC_) of 1.01 V, a short-circuit current of 21.0 mA/cm^2^, a fill factor (*FF*) of 76.0%, and a *PCE* of 14.6% ± 1.5%.^2^ Additionally, when using NiO_x_ as the HTL instead of poly(3,4-ethylenedioxythiophene):polystyrene sulfonate (PEDOT:PSS), which is acidic and hygroscopic in nature, the stability of PSCs was greatly improved.

Self-assembled monolayers (SAMs) have emerged as a versatile approach for modifying the interfacial layer of PSCs, owing to their excellent features, e.g., ease of processing, low material consumption, compatibility with various substrates, and efficient charge extraction.

Phosphoric-acid-based SAMs are bonded to the substrate through phosphoric acid functionality as an anchoring group and feature common π-functional tail moieties such as carbazole, acridine, and phenyl groups and their derivatives. These tail moieties are linked to the anchoring groups through an alkyl spacer, playing crucial roles in hole extraction and surface passivation for perovskite active materials. Notably, a SAM incorporating 7*H*-dibenzocarbazole as the tail group was able to enhance the performance of all-perovskite tandem solar cells with an aperture area of 1.044 cm^2^, achieving a notable *PCE* of 27.0%.^3^

## Mechanism

At the same time, the thermal stability of SAMs remains inadequate for outdoor solar modules, and the sparse and inhomogeneous SAMs may result in high leakage current at the interface. In contrast, NiO_x_, as a p-type semiconductor, has the potential to reduce interfacial charge recombination and facilitate rapid hole collection in PSCs by forming dense films with high hole mobility. However, the significant surface roughness of NiO_x_ films produced by solution processes hinders the high-quality crystallization of perovskite. Unlike NiO_x_, SAMs interact with the perovskite surface via tail groups, which can promote the nucleation and growth of more perfect perovskite crystals. This interaction reduces the trap density on the perovskite surface, leading to the higher *FF* and *V*_OC_ of PSCs.^4^

## Photovoltaic modules

Recently, You’s group reported that anchoring phosphonic-acid-based SAM ([4-(3,6-dimethyl-9*H*-carbazol-9-yl)butyl]phosphonic acid [Me-4PACz]) to a thin layer of NiO_x_ homogenized nanoparticles as an HTL by spin coating efficiently enhanced hole extraction, reduced interfacial recombination, and improved the operational stability of PSCs.^5^ A p-type NiO_x_ layer is required to ease SAM deposition and minimize charge recombination and also facilitate better charge transport. To achieve this, the authors of the study developed an efficient method for modulating NiO_x_ nanoparticles using treatment with H_2_O_2_. This treatment led to an increased ratio of Ni^3+^ in NiO_x_ and the formation of NiOOH (likely from Ni(OH)_2_), which is a stable component with high conductivity. The presence of NiOOH also introduced more surface hydroxyl groups for SAM binding. Additionally, the treatment prevented NiO_x_ nanoparticle aggregation and reduced the nanoparticle size (from ∼10 to ∼5 nm). Consequently, spin coating resulted in denser and more uniform NiO_x_ films. This method succeeded in dispersing NiO_x_ nanoparticles in deionized water, which provides an economic and environmentally friendly alternative to using organic HTLs that require deposition from organic solvents.

**Figure 1:**
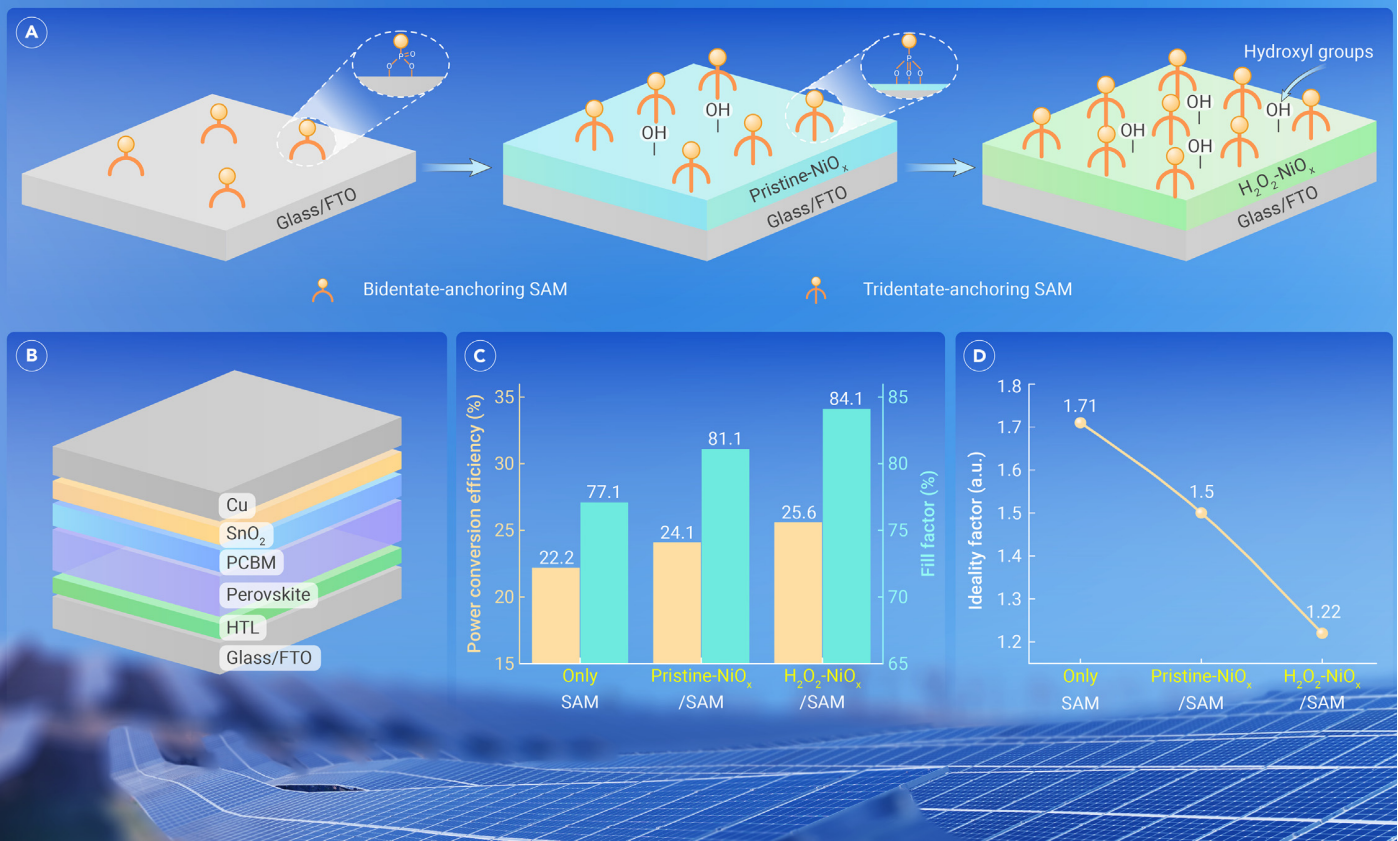
Fabrication and performance of perovskite solar cells (A) Schematic diagrams of fluorine-doped tin oxide (FTO) anode modified with different hole extraction layers (SAM, pristine-NiO_x_/SAM, H_2_O_2_-NiO_x_/SAM). (B) Device structure of the inverted perovskite solar cell. (C) Comparison of power conversion efficiency and fill factors of the devices. (D) Diode ideality factors of the devices.

The PSCs with different HTLs (see Figures 1A and 1B), i.e., Me-4PACz SAM, pristine-NiO_x_/Me-4PACz SAM, and H_2_O_2_-NiO_x_/Me-4PACz SAM, respectively, were constructed and compared. It has been reported that NiO_x_ would be reactive with halide perovskite. When depositing SAMs on NiO_x_, an increment in the number of surface hydroxyl groups promotes the formation of P=O⋯Ni bonding, leading to tridentate anchors. SAM itself shows inferior thermal stability compared to inorganic hole-extracting analogs. By combining SAM and pristine-NiO_x_, the problems of poor interfacial thermal stability and direct contact of perovskite and NiO_x_ are avoided. Compared with the devices with the SAM-only HTL, the *PCE* and *FF* of the pristine-NiO_x_-based PSC were increased to 24.1% and 81.1%, respectively. After modulating the structure of NiO_x_ nanoparticles with H_2_O_2_, even higher *PCE* (25.6%) and *FF* (84.1%) were simultaneously obtained (Figure 1C). Meanwhile, the diode ideality factor of PSCs was modified from 1.71 to 1.22 (Figure 1D). It is very encouraging that 85.4% of the initial *PCE* was retained after 1,000 h of continuous operation at 50°C (85.1% after 500 h at 85°C). A fabricated PSC minimodule (6 subcells of 14.6 cm^2^ in total, *V*_OC_ = 6.9 V) demonstrated a *PCE* of 21%, which is among the highest efficiencies for large-area PSCs.

## Concluding remarks

To improve the performance of PSCs, interface engineering is crucial, as it passivates perovskite structure defects, reduces interfacial charge recombination, and thus enhances hole extraction and carrier transport. Transition metal oxides, like NiO_x_, MoO_x_, and WO_x_, are more chemically stable than PEDOT:PSS, offering superior operational stability. However, challenges remain with transition metal oxides related to carrier extraction efficiency, chemical stability, and defect control. A promising approach is to cover metal oxide nanoparticle layers with SAMs to facilitate proper nucleation and growth of the perovskite layer over the HTL. The development of efficient and versatile SAM candidates is required to simplify the fabrication process and enhance device performance while addressing issues related to film formation and uniform coverage of the substrate surface. By combining the advantages of SAMs and metal oxides, high-performance and stable large-area PSCs should be developed for commercial applications. This strategy would be applicable to other solution-processed optoelectronic devices such as organic photodiodes, organic solar cells, organic light-emitting diodes, and even organic lasers.
